# Myc derived circRNA promotes triple-negative breast cancer progression via reprogramming fatty acid metabolism

**DOI:** 10.1007/s12672-023-00679-2

**Published:** 2023-05-12

**Authors:** Shengting Wang, Yufang Wang, Yue Wang, Qian Li, Kaixuan Zeng, Xiaoming Li, Xinghua Feng

**Affiliations:** 1 Department of Clinical Medicine, Xi’an Peihua University, 888 Changning Street, Xi’an, Shaanxi 710125 China; 2 Department of Gastroenterology, The First Affiliated Hospital of Nanchang University, Nanchang, 330006 China; 3 School of Medicine, Xi’an Jiaotong University, Xi’an, 710000 China

**Keywords:** CircRNA, Metabolic reprogramming, Triple-negative breast cancer

## Abstract

Myc is a well-known proto-oncogene that is frequently amplified and activated in breast cancer, especially in triple-negative breast cancer (TNBC). However, the role of circular RNA (circRNA) generated by Myc remains unclear. Herein, we found that circMyc (hsa_circ_0085533) was remarkably upregulated in TNBC tissues and cell lines, which was attributed to gene amplification. Genetic knockdown of circMyc mediated by lentiviral vector significantly inhibited TNBC cell proliferation and invasion. Importantly, circMyc increased cellular triglycerides, cholesterols and lipid droplet contents. CircMyc was detected in both cytoplasm and nucleus, cytoplasmic circMyc could directly bind to HuR protein, facilitating the binding of HuR to SREBP1 mRNA, resulting in increasing SREBP1 mRNA stability. Nuclear circMyc bound to Myc protein, facilitating the occupation of Myc on SREBP1 promoter, leading to increasing SREBP1 transcription. As a result, the elevated SREBP1 increased the expression of its downstream lipogenic enzymes, enhancing lipogenesis and TNBC progression. Moreover, the orthotopic xenograft model showed that depletion of circMyc markedly inhibited lipogenesis and reduced tumor size. Clinically, high circMyc was closely related to larger tumor volume, later clinical stage and lymph node metastasis, functioning as an adverse prognostic factor. Collectively, our findings characterize a novel Myc-derived circRNA controlling TNBC tumorigenesis via regulation of metabolic reprogramming, implying a promising therapeutic target.

**Supplementary Information:**

The online version contains supplementary material available at 10.1007/s12672-023-00679-2.

## Introduction

Breast cancer is a phenomenon of uncontrolled proliferation of breast epithelial cells under the action of a variety of carcinogens [1]. Its incidence rate ranks first among female malignant tumors, while male breast cancer is relatively rare [2]. Triple-negative breast cancer (TNBC) refers to breast cancer with negative estrogen receptor (ER), progesterone receptor (PR) and proto-oncogene Her-2, accounting for 15–20% of all pathological types of breast cancer, with a special biological behavior and clinical pathological characteristics [3]. Due to the lack of effective therapeutic targets, the long-term efficacy of TNBC is not desirable, with a dismal 5-year survival prognosis [4]. Therefore, clarifying the potential mechanism of TNBC is of great significance for developing new therapies targeting this thorny disease.

Circular RNA (circRNA) is a special class of non-coding RNA, characterized by the closed loop structure [5]. It is extremely stable in biological cells and is a hot target for treating human diseases[6]. Some circRNAs with specific functions can be used as biomarkers for disease diagnosis, and also as the therapeutic targets for a number of human diseases, especially immune diseases and cancers [7]. The generation, structure and degradation mechanism of circRNA are different from that of mRNA, giving rise to the unique cellular functions of circRNA [8]. Recent evidence suggests that circRNA has multiple modes of action, including regulation of transcription, splicing and chromatin interactions, acting as a microRNA decoy and protein scaffold, and even directly translating into functional peptides [9]. Data from us and other laboratories reveal the intrinsic linking between circRNA and breast cancer [10], for example, circBGN was upregulated in HER2-positive breast cancer and conferred trastuzumab resistance through repressing SLC7A11-mediated ferroptosis [11]; circTRIO was identified as a potential oncogenic regulator in TNBC, which regulated the miR-432-5p/CCDC58 axis [12]. Although a small number of circRNAs have been found to be associated with TNBC, a large proportion of their functions are unknown and need further elucidation.

CircRNA is generated from pre-mRNA reverse splicing, thus one gene locus can generate multiple circRNAs through variable reverse splicing via altering the selection of splicing sites [13]. Studies have shown that some circRNAs regulated their parental gene expression or functions, participating in human disease progression [14]. For instance, circFOXO3 was shown to protect against osteoarthritis by activating autophagy via targeting its parental gene FOXO3 [15]; circYAP inhibited oncogenic YAP translation by suppressing the assembly of the translation initiation machinery [16]. Myc, the well-documented proto-oncogene, is frequently overexpressed in various human cancers [17]. Accumulated evidence shows that Myc is involved in multiple signaling pathways controlling cell growth, differentiation, stemness, metastasis, etc [18]. Not surprisingly, Myc is significantly increased in breast cancer, especially in TNBC [19]. However, the expression and function of circRNA derived from Myc in TNBC, and whether they affect the role of Myc, are unknown.

In this study, we tested the levels of Myc-derived circRNAs in breast cancer, and found that circMyc (hsa_circ_0085533) was remarkably elevated in TNBC. Further investigations showed that circMyc promoted TNBC progression via regulating fatty acid metabolism in a Myc-dependent and -independent manner.

## Materials and methods

### Tissue samples

A total of 15 adjacent normal tissues (> 3 cm from the edge of the tumor) and 96 breast cancer tissues (70 cases of non-TNBC, 26 cases of TNBC) were collected from The Affiliated Shaanxi Fourth People Hospital of Peihua University. The patients have not received any anti-tumor treatment, and all tissues have been confirmed as breast cancer by two independent pathologists. The detailed clinicopathological information is shown in Supplementary Table S1. The protocol of collecting specimen was approved by the Ethics Committee of The Affiliated Shaanxi Fourth People Hospital of Peihua University, which was conducted in compliance with the guidelines of the Declaration of Helsinki.

### Cell lines and transfection

The normal mammary epidermal cells MCF-10 A and six breast cancer cells (MCF-7, T47D, SK-BR-3, BT-20, MDA-MB-231 and MDA-MB-468) were obtained from ATCC (Manassas, USA) and cultured in DMEM medium supplemented with 10% fetal bovine serum (FBS). Mycoplasma contamination was detected every three months. All cells used for experiments were no more than ten passages. Cell transfection was performed using Lipofectamine 3000 (Invitrogen, CA, USA) as per the manufacturer’s instructions. In brief, BT-20 and MDA-MB-231 cells were plated onto 6-well plate. After cells reached to be 70–80% confluent, 5µL Lipofectamine 3000 reagent pre-mixed with 125 µL opti-MEM was added into cells, followed by addition with 2.5 µg plasmids (pcDNA 3.0 (control empty vector), pcDNA-Myc, pcDNA-SREBP1 or pcDNA-HuR) pre-mixed with 125 µL opti-MEM. After 48 h of transfection, cells were collected for the subsequent assays. Cell transfection was conducted in triplicate with three independent biological experiments.

### Immunohistochemistry (IHC) and western blot

26 cases of TNBC tissues were made into paraffin-embedded blocks, which were then cut into 4 μm sizes for staining. After antigen retrieval, the slides were incubated with recombinant anti-c-Myc antibody (ab32072, Abcam) overnight at 4℃. The next day, the slides were incubated with HRP Substrate Chromogenic DAB kit (CWBIO, Beijing, China). The IHC staining results were analyzed by two independent experienced pathologists. Interpretation of IHC staining was determined by using the semiquantitative H-score method as previously described [20]. Staining percentages (0–100%) and staining intensity of Myc (0–3: 0, negative; 1, very weak; 2, moderate; 3, strong) were recorded, and the H-score values were calculated (0–300) by multiplying staining percentages by staining intensity, the maximum H-score value is 300. Western blot was carried out with the standard protocols. In brief, cell lysate was collected and boiled for 10 min. A total of 20 µg protein was loaded onto SDS-PAGE gel, followed by transfer onto PVDF membrane. After blocking with 5% non-fat milk powder for 1 h at room temperature, the PVDF membrane was incubated with anti-SREBP1 antibody (ab191857) at 4 °C overnight. The next day, the PVDF membrane was incubated with HRP-conjugated secondary antibody, followed by development using the Enhanced ECL Chemiluminescence Detection Kit (Ready-to-use) (Vazyme, Nanjing, China). Western blot was conducted in triplicate with three independent biological experiments.

### qRT-PCR assay

Total RNA was extracted from fresh tissues and cell lines using 1mL Trizol solution (Invitrogen) at room temperature for 5 min. Then, 200µL chloroform was added, followed by shake vigorously with hands for 15s and centrifugation at 12,000 g for 15 min. The upper aqueous phase was collected, added with 500µL isopropanol and centrifuged at 12,000 g for 15 min. RNA was precipitated at the bottom of the centrifuge tube and washed with 1mL 75% ethanol. Then, RNA was dissolved in RNase-free water. RNA quantification was conducted using NanoDrop™ Lite spectrophotometer (Invitrogen). Then, the PrimeScriptRT Master Mix (Takara Bio, Otsu, Japan) was used to synthesize cDNA with 1 µg RNA, followed by quantification with SYBR PremixEx Taq Kit (Takara Bio). At least three repeat wells were set for all detected genes, and the relative gene expression to GAPDH was analyzed by 2^−ΔΔCt^ method. qRT-PCR was repeated with three independent biological experiments. The primer sequences used in this study are shown below:

Circ-0085533: Forward: 5`-GCTGCTTAGACGCTGGATTT-3`, Reverse: 5`-AGAAGCCCTGCCCTTCTC-3`;

Circ-0085534: Forward: 5`-AAGTACATTTTGCTTTTTAAAGTTGA-3`, Reverse: 5`-AGAAGCCCTGCCCTTCTC-3`;

Circ-0085535: Forward: 5`-GGCAAATATATCATTGAGCCAAA-3`, Reverse: 5`-CCTCCTCGTCGCAGTAGAAA-3`;

FASN: Forward: 5`-CACTGGACACAGCCTGCTC-3`, Reverse: 5`-CTCAAGAACTGCACGGAGGT-3`;

ACLY: Forward: 5`-GATCCCCATCCATGTCTTTG-3`, Reverse: 5`-TGAGGAGGAAGTTTGCAGTG-3`;

ACC: Forward: 5`-TTCAAGCTGAAGTTCCTGGAT-3`, Reverse: 5`-CTTCTGATGCCTGCGTTGTA-3`;

SCD: Forward: 5`-TCCTGCAGAATGGAGGAGAT-3`, Reverse: 5`-CATACAGGGCTCCCAAGTGT-3`;

chREBP: Forward: 5`-GGAGCAATGGTGCAAACAG-3`, Reverse: 5`-CGGACTGAGTCATGGTGAAG-3`;

SREBP1: Forward: 5`-GCACCCACTCCATTGAAGAT-3`, Reverse: 5`-TAGCCTAACACAGGGGTGGA-3`;

SREBP2: Forward: 5`-TGGCAGTGGTGGTAGTGGTA-3`, Reverse: 5`-TGGGAGAAACCTTGACTTGC-3`;

Myc: Forward: 5`-CCCTCAACGTTAGCTTCACC-3`, Reverse: 5`-CAGCAGCTCGAATTTCTTCC-3`;

GAPDH: Forward: 5`-ACCCAGAAGACTGTGGATGG-3`, Reverse: 5`-TTCAGCTCAGGGATGACCTT-3`.

### Lentivirus infection

Three shRNAs (sh-circMyc#1: 5`-GTAGTGGAAAACCAGGACCCC-3`; sh-circMyc#2: 5`-AAACCAGGACCCCCGAGCTGT-3`; sh-circMyc#2: 5`-GAAAACCAGGACCCCCGAGCT-3`) targeting the junction site of circMyc was designed by CircInteractome online tool [21]. Then, the synthesized sequences were inserted into psi-LVRU6GP lentiviral vector (GeneCopoeia, CA, USA) and transfected into 293T cells using Lipofectamine 3000. After 48 h, the virus particles were collected and infected into BT-20 and MDA-MB-231 cells with polybrene. The stable circMyc-silenced cell lines were screened by puromycin and tested by qRT-PCR analysis.

### The functional assays

Cell viability was tested by CCK-8 assay using CCK-8 solution (#HY-K0301, MedChemExpress). In brief, BT-20 or MDA-MB-231 cells were seeded onto 6-well plate and cultured for 24 h, 48 and 72 h at 37 °C. Then, 10 µL CCK-8 reagent was added into each well, followed by incubation for 2 h at 37 °C. The light absorption value at 450 nm in each was recorded. The ratio of DNA synthesis was detected by EdU Cell Proliferation Kit with Alexa Fluor 555 (Epizyme, Shanghai, China) as per to the manufacturer’s instructions. In brief, BT-20 or MDA-MB-231 cells were seeded onto 96-well plate, followed by addition with 10µM EdU reagent and incubation for 2 h at 37 °C. The supernatant was discarded and cells were fixed with 1 mL 4% polyformaldehyde for 15 min at room temperature. After three times of washing with PBS, cells were incubated with 430µL Click Reaction Buffer supplemented with 20 µL CuSO_4_, 1 µL 555 Azide and 50 µL Click Additive Solution for 30 min at room temperature. Then, the above solution was discarded and cells were incubated with 1mL DAPI solution for 10 min at room temperature. The EdU positive cells were observed under fluorescence microscope. Cell invasion was tested by Transwell assay using Transwell chamber (Corning, CA, USA). In short, the Transwell chamber coated with Matrigel (Corning) was mounted on 24-well plate, then, 200µL DMEM medium containing 5 × 10^4^ BT-20 or MDA-MB-231 cells were seeded onto the upper Transwell chamber, while the lower chamber was added with 600µL DMEM medium supplemented with 10% FBS. After 24 h of incubation at 37 °C, the invaded cells were fixed with 1mL 4% polyformaldehyde for 15 min at room temperature, followed by staining using 0.1% crystal violet. The stained cells were photographed and counted in five randomly selected fields under a microscope. The above functional assays were repeated independently for three times, and each group was set with three technical repeat wells.

### Nile red staining, triglyceride and cholesterol assays

BT-20 and MDA-MB-231 cells were plated on 96-well plate and were grown to 70–80% confluence. The culture medium was discarded and cells were washed by PBS and fixed by 4% paraformaldehyde solution for 15 min at room temperature. Then, cells were stained with 5 µg/mL Nile Red solution (MedChemExpress) for 15 min at room temperature, and then stained with DAPI. The images were visualized by immunofluorescence microscopy. For detection of triglyceride content, the Tissue &Cell Triglyceride Content Enzymatic Determination kit (E1013-50, Applygen Technologies Inc., Beijing, China) was used. In brief, 2 × 10^6^ BT-20 or MDA-MB-231 cells were washed with PBS and lysed using 100 µL lysis buffer at room temperature for 10 min. Then, the lysis was transferred to the 1.5 mL centrifuge tube, 90 µL lysis was used for protein quantification, and the remaining 10 µL lysis was incubated at 70 °C for 10 min, followed by centrifugation at 2000 rpm for 5 min. The supernatant was incubated with reaction buffer at 37 °C for 15 min. The triglyceride content was calculated based on multiple dilution of 4mM glycerol standard and justified based on protein content. For testing cholesterol levels, the Tissue&Cell Total Cholesterol Content Enzymatic Determination kit (E1015-50, Applygen Technologies Inc.) was used. 2 × 10^6^ BT-20 or MDA-MB-231 cells in 6-well plate were lysed using 200 µL lysis buffer at room temperature for 10 min. Then, 10 µL lysis was incubated with reaction buffer at 37 °C for 20 min. The cholesterol content was calculated based on multiple dilution of 5mM cholesterol standard using anhydrous ethanol and justified based on protein content. The above assays were repeated independently for three times, and each group was set with three technical repeat wells.

### RNA pull-down and RNA immunoprecipitation (RIP) assays

The protocols for RNA pull-down and RIP were shown in our previous study [11]. In brief, the endogenous probe used for pull-down assay was designed according to the junction site of circMyc (5`-TGCTTAGACGCTGGATT-3`), followed by labeled with biotin. Then, the probes were incubated with 500µL collected lysates containing 1 × 10^7^ BT-20 or MDA-MB-231 cells at 4 °C overnight with rotation. The next day, the lysates were incubated with 100µL Pierce™ Streptomycin Avidin Agarose (20,349, Invitrogen) at 4 °C with rotation for 3 h. After washing with lysis buffer six times, the proteins enriched on agarose were eluted by 1×SDS loading buffer and boiled for 5 min, followed by western blot analysis of HuR and Myc levels using anti-HuR (ab200342, Abcam) and anti-c-Myc (ab32072, Abcam) antibodies, respectively. For RIP assay, 1 × 10^7^ BT-20 or MDA-MB-231 cells were washed by pre-cooled PBS twice and lysed using 500µL lysis buffer. Then, 100 µL Pierce™ Protein A/G agarose magnetic beads (78,610, Invitrogen) and 5 µg anti-Myc (ab32072, Abcam) or anti-HuR (ab200342, Abcam) were added into the lysates, and incubated overnight at 4 °C with rotation. After washing with lysis buffer six times, the beads were resuspended with 150 µL Proteinase K buffer, followed by incubation at 55 °C for 30 min. Then, the beads were resuspended by 1mL Trizol reagent, followed by qRT-PCR analysis. The above assays were repeated independently for three times, and each group was set with three technical repeat wells.

### Luciferase reporter assay

BT-20 or MDA-MB-231 cells were plated onto 35 mm cell culture dish, and after cells grow to 70–80% confluence, the pGL3-Basic vector (Promega, WI, USA) containing SREBP1 promoter sequence with Myc binding motif (− 919 to − 908) was transfected into cells with a Renilla luciferase plasmid at a ratio of 10:1 using Lipofectamine 3000. After 36 h of transfection, cells were washed by pre-cooled PBS twice, and collected by 350µL pre-cooled harvest buffer (0.05 M Tris-HCl (pH = 7.5), 1mM DTT, 0.1% Triton X-100). The Dual-Luciferase Reporter Assay System (Promega) was used to test the luciferase activity according to the manufacturer’s instructions. The above assays were repeated independently for three times, and each group was set with three technical repeat wells.

### Chromatin immunocoprecipitation (ChIP)

The ChIP assay was performed using Simple ChIP Enzymatic Chromatin IP Kits (9003, Cell Signaling Technology) according to manufacture instructions. In brief, BT-20 or MDA-MB-231 cells were plated onto 10 cm cell culture dish, and after cells grow to 90–100% confluence, cells were washed by pre-cooled PBS twice, and treated with 1% formaldehyde for 10 min at room temperature to cross-link proteins and DNA. The formaldehyde was inactivated by glycine for 5 min at room temperature. Then, 1 µL micrococcal nuclease was added into the lysates and incubated at 37 ℃ for 20 min to digest the DNA into a length of about 150–900 bp, during which it was turned over every three to five minutes for mixing. 5 µg anti-Myc (ab32072, Abcam) or IgG antibody was added into above lysates and incubated at 4℃ for 4 h, followed by incubation with 50 µL ChIP-Grade Protein G Magnetic Beads (9006, Cell Signaling Technology)at 4 ℃ for 2 h. After washing, the beads were incubated with 150µL 1×ChIP Elution Buffer at 65 ℃ for 30 min. Then, 6 µL 5 M NaCl and 2µL Proteinase K were added and incubated at 65 ℃ overnight. The enriched DNA was purified by QIAamp DNA Mini Kit (51,304, QIAGEN) according to the manufacturer’s instructions, followed by qPCR analysis of the enrichment of SREBP1 promoter using the following primer: Forward: 5`-AGGGTGCTTCTCTCTGCTTG-3`, Reverse: 5`-CTGTTGAGAAGAGGGCTGGA-3`.

### Orthotopic tumor model

All animal studies were performed following institutional guidelines of the Animal Care and Use Committee of Peihua university. The protocol was described in our previous study [11]. In brief, the female NOD/SCID mice aged 4–6 weeks and weighing 18-22 g were anesthetized using isoflurane, then, 2 × 10^6^ BT-20 and MDA-MB-231 cells suspended in 200 µL pre-cooled PBS were inoculated orthotopically onto the abdominal mammary fat pad, which were grown under specific pathogen free (SPF) conditions. Tumor volume was recorded once a week with a vernier caliper. After four weeks, all mice were sacrificed, tumor weight was recorded and tumor samples were collected for qRT-PCR analysis. Extensive efforts were made to ensure minimal suffering of the mice used in this study.

### Statistical analysis

The data were the mean ± standard deviation (SD) of at least three independent experiments carried out in triplicate. The two-tailed Student’s t-test was used to compare the differences between the two groups. Multi-group comparison was performed using one-way or two-way ANOVA with Tukey post-hoc test. The survival probability was tested by Kaplan-Meier plotter and analyzed by log-rank test. All data were analyzed by SPSS or GraphPad Software. *P* < 0.05 was considered statistically significance. *P < 0.05, **P < 0.01, ***P < 0.001.

## Results

### CircMyc is highly expressed in TNBC

**Fig. 1:**
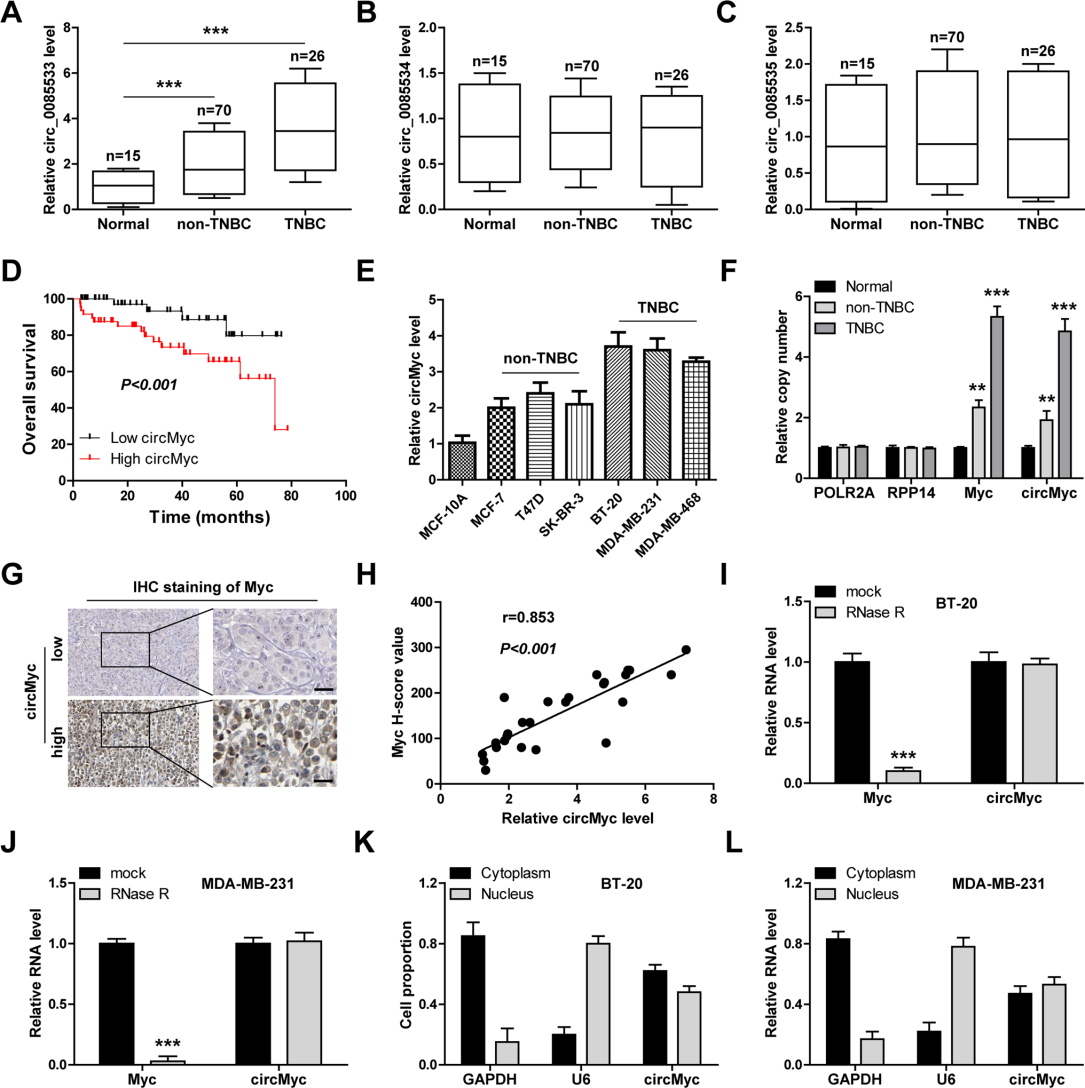
CircMyc is increased in TNBC. **A–C** qRT-PCR analysis of expression of these three Myc-derived circRNAs (hsa_circ_0085533, hsa_circ_0085534 and hsa_circ_0085535) in adjacent normal (n = 15), non-TNBC (n = 70) and TNBC (n = 26) tissues. Gene relative expression was calculated using 2^−ΔΔCt^ method, and GAPDH was used as reference control. Data were shown as boxplot contianing median ± quartile deviation, and analyzed by one-way ANOVA with Tukey post-hoc test. **D** The survival curve of breast cancer patients in our cohort (non-TNBC, n = 70; TNBC, n = 26) with low (n = 45) and high (n = 45) circMyc expression. The median circMyc expression value was used to define low and high circMyc groups. Data was analyzed by log-rank test. **E** qRT-PCR analysis of circMyc expression in normal MCF-10 A cells, non-TNBC cells (MCF-7, T47D, SK-BR-3) and TNBC cells (BT-20, MDA-MB-231, MDA-MB-468). Gene relative expression was calculated using 2^−ΔΔCt^ method, and GAPDH was used as reference control. Data were shown as the mean ± SD of at least three independent experiments carried out in triplicate. Data were analyzed by one-way ANOVA with Tukey post-hoc test. **F** Analysis of copy number of Myc and circMyc in adjacent normal (n = 15), non-TNBC (n = 70) and TNBC (n = 26) tissues. POLR2A and RPP14 were used as reference controls. Data were shown as mean ± SD, and analyzed by one-way ANOVA with Tukey post-hoc test. **G** IHC staining of Myc protein in TNBC tissues (n = 26), Myc was stained in cell nucleus, and brown is positive staining. Scale bar, 50 μm. H. The correlation between circMyc and Myc protein expression in TNBC tissues (n = 26). Data was shown as scatter diagram and analzyed by spearman correlation coefficient. **I**, **J** qRT-PCR analysis of circMyc and Myc expression in BT-20 and MDA-MB-231 cells treated with 3U/µg RNase R at 37 ℃ for 20 min. Data were shown as the mean ± SD of at least three independent experiments carried out in triplicate. Data were analyzed by unpaired Student’s t-test. **K**, **L** qRT-PCR analysis of circMyc location in BT-20 and MDA-MB-231 cells, GAPDH and U6 were used as cytoplasmic and nuclear control fragments, respectively. Data were shown as the mean ± SD of at least three independent experiments carried out in triplicate. Data were analyzed by unpaired Student’s t-test. TNBC, triple-negative breast cancer; SD, standard deviation. Two-tailed **P < 0.01, ***P < 0.001

Through analyzing CircBase database [22], we found that Myc is able to generate three circRNAs (Fig. S1). Then, we tested their expression levels in breast cancer tissues, as shown in Fig. 1A–C, only circMyc (hsa_circ_0085533) was significantly upregulated in breast cancer, especially in TNBC. CircMyc expression was positively correlated with tumor size, lymph node metastasis and TNM stage (Table 1). Moreover, patients with high circMyc had an adverse overall survival time (Fig. 1D). Likewise, high circMyc was further observed in breast cancer cell lines, especially in TNBC cells (Fig. 1E). Through analyzing TCGA data using cBioPortal online tool (Fig. S2), we found that Myc gene was frequently amplified in breast cancer (15.01–40.11%), especially in the invasive subtype. Thus, we inferred that circMyc upregulation might be the result of Myc gene amplification. As expected, the copy numbers of Myc and circMyc were both increased in breast cancer tissues, especially in TNBC cases (Fig. 1F). And the expression of circMyc was strongly positively linked with Myc expression in TNBC tissues (Fig. 1G, H). In addition, we test the attribute of circMyc, found that circMyc, but not Myc mRNA, was extremely resistant to RNase R (Fig. 1I, J). And circMyc was expressed in both cytoplasm and nucleus (Fig. 1K, L).

**Table 1:** Association of circMyc expression with clinical parameters in 96 breast cancer patients

Parameters	Total (n = 96)	circMyc expression		*p* value
		Low (n = 48)	High (n = 48)	
Age (years)				
≤ 40	25	13	12	0.816
> 40	71	35	36	
Menopause				
Yes	42	19	23	0.411
No	54	29	25	
Tumor size (cm)				
≤ 2	33	22	11	0.018
> 2	63	26	37	
LN metastasis				
Negative	44	31	13	<0.001
Positive	52	17	35	
TNM stage				
I	24	18	6	0.006
II	43	21	22	
III	29	9	20	
Histological Grade				
I	19	9	10	0.444
II	42	24	18	
III	35	15	20	

*LN* Lymph node

### CircMyc silencing inhibits TNBC cell proliferation and invasion

**Fig. 2:**
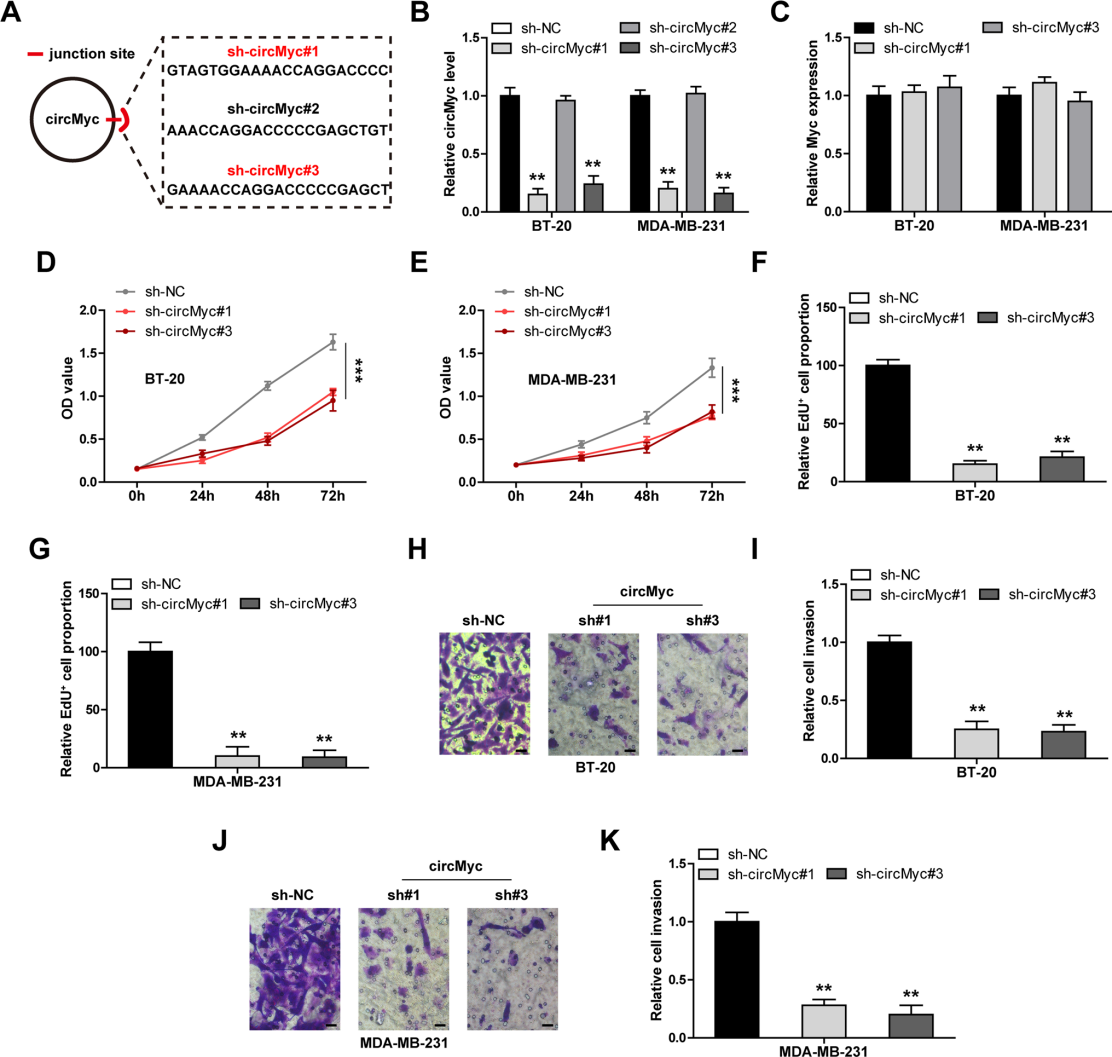
Knockdown of circMyc inhibits TNBC cell malignant phenotype. **A** The diagrammatic sketch showing three designed shRNAs targeting circMyc junction sites. **B**, **C** qRT-PCR analysis verifying the knockdown efficiency of circMyc by above designed shRNAs in BT-20 and MDA-MB-231 cells. Data were shown as the mean ± SD of at least three independent experiments carried out in triplicate. Data were analyzed by one-way ANOVA with Tukey post-hoc test. **D**, **E** CCK-8 assay testing cell viability in control and circMyc-silenced BT-20 and MDA-MB-231 cells at 24 h, 48 and 72 h using an automatic microplate spectrophotometer. Data were shown as the mean ± SD of at least three independent experiments carried out in triplicate. Data were analyzed by two-way ANOVA with Tukey post-hoc test. **F**, **G** EdU staining testing DNA synthesis rate of BT-20 and MDA-MB-231 cells with or without circMyc knockdown. The stained cells were photographed and counted in five randomly selected fields under a microscope. Data were shown as the mean ± SD of at least three independent experiments carried out in triplicate. Data were analyzed by one-way ANOVA with Tukey post-hoc test. **H–K** Transwell assay using chamber testing cell invasion in BT-20 and MDA-MB-231 cells with or without circMyc knockdown. The invaded cells were stained by crystal violet, were photographed and counted in five randomly selected fields under a microscope. Data were shown as the mean ± SD of at least three independent experiments carried out in triplicate. Data were analyzed by one-way ANOVA with Tukey post-hoc test. Scale bar, 25 μm. TNBC, triple-negative breast cancer; SD, standard deviation. Two-tailed **P < 0.01, ***P < 0.001

Three shRNAs were designed to study the functions of circMyc (Fig. 2A). As shown in Fig. 2B, the sh-circMyc#2 had no effect on knocking down circMyc. Thus, we chose sh-circMyc#1 and #3 for the subsequent assays. And Myc levels were unaffected by sh-circMyc#1 and #3, suggesting that these shRNAs specifically targets circMyc (Fig. 2C). The CCK-8 results showed that silencing of circMyc reduced TNBC cell viability (Fig. 2D, E). Further, less EdU^+^ cells were observed in circMyc-silenced group as compared to control group (Fig. 2F, G). The results of Transwell assay showed that the invasiveness of TNBC cells was significantly reduced after circMyc knockdown (Fig. 2H–K).

### Knockdown of circMyc significantly reduces lipid contents in TNBC cells

**Fig. 3:**
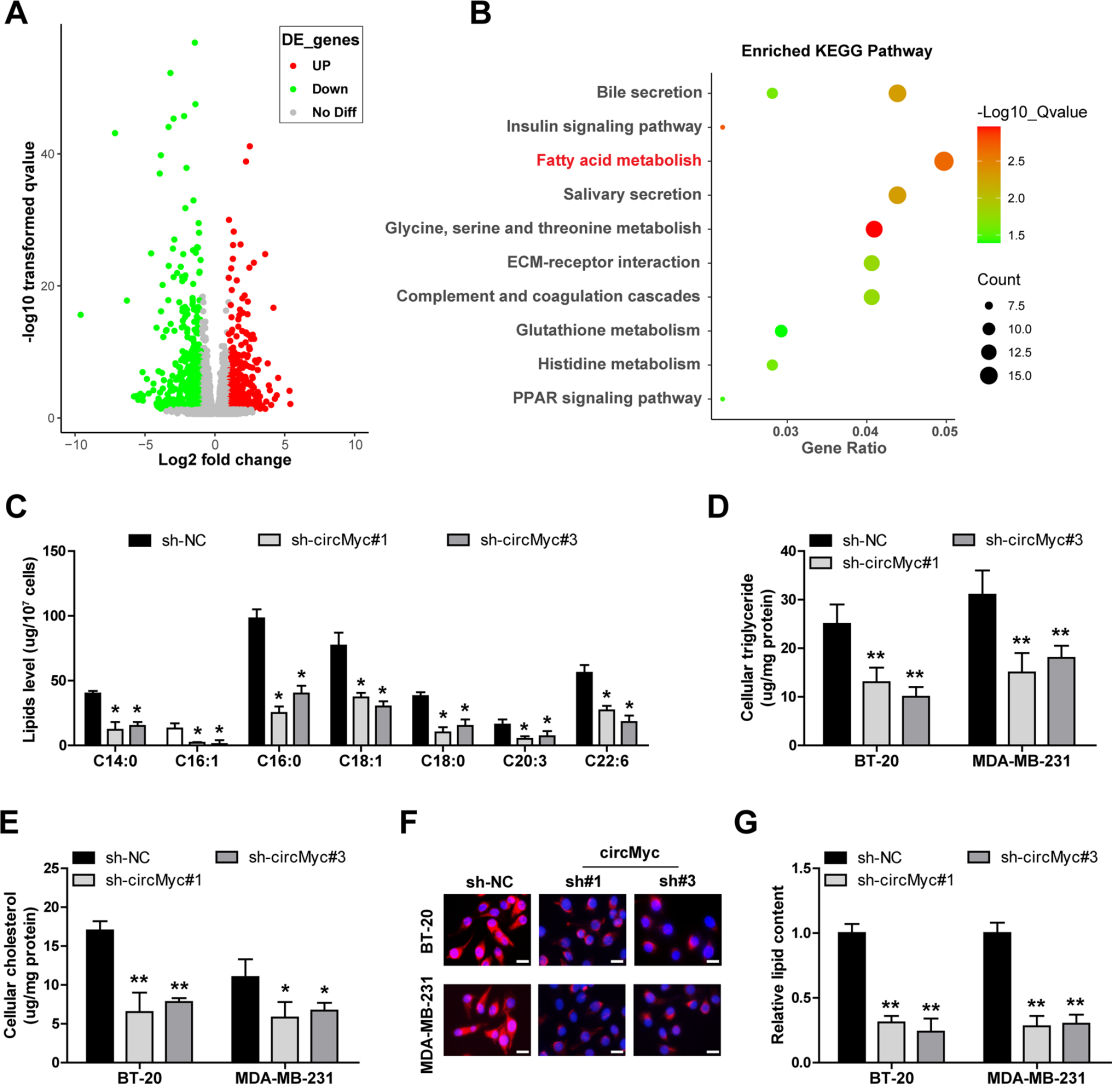
CircMyc is involved in fatty acid metabolism. **A** 1 × 10^7^ BT−20 cells with circMyc knockdown (n = 3) and control cells (n = 3) were washed by PBS and treated with Trizol reagent, followed by RNA sequencing. The differentially expressed genes were defined as *P* value of FDR < 0.05 and |log2 (fold change)|>1, which was analyzed by using RStudio with DESeq2 package. Red denotes upregulated genes, green denotes downregulated genes. **B** KEGG pathway enrichment analysis of the differentially expressed genes was conducted by using RStudio with ClusterProfiler package. X axis denotes the proportion of the differentially expressed genes enriched to the target pathway; Y axis denotes specific pathway; The size of the bubble denotes the number of genes, larger size denotes more genes; The color of bubble denotes the significance of enrichment, red denotes higher significance, and green denotes lower significance. **C** Gas chromatography-mass spectrometry analysis of free fatty acids in control and circMyc-silenced BT-20 cells. Data were shown as the mean ± SD of at least three independent experiments carried out in triplicate. Data were analyzed by one-way ANOVA with Tukey post-hoc test. **D**, **E** The effects of circMyc silencing on triglyceride and cholesterol levels were tested by Enzymatic Determination kit, and the triglyceride and cholesterol levels were calculated based on multiple dilution of standard and justified based on protein content. Data were shown as the mean ± SD of at least three independent experiments carried out in triplicate. Data were analyzed by one-way ANOVA with Tukey post-hoc test. **F**, **G** Nile Red staining testing lipid contents in control and circMyc-silenced BT-20 and MDA-MB-231 cells. Red denotes positive Nile staining, and cell nucleus was stained by DAPI (blue). The stained cells were photographed and counted in five randomly selected fields under a microscope. Data were shown as the mean ± SD of at least three independent experiments carried out in triplicate. Data were analyzed by one-way ANOVA with Tukey post-hoc test. Scale bar, 25 μm. SD, standard deviation. Two-tailed *P < 0.05, **P < 0.01

To explore the potential mechanism of circMyc, we conducted RNA sequencing in circMyc-silenced BT-20 cells (Fig. 3A). The enriched KEGG pathway analysis showed that circMyc was closely linked to fatty acid metabolism (Fig. 3B). The contents of free fatty acids were dramatically decreased in circMyc-silenced TNBC cells, such as palmitic acid and oleic acid (Fig. 3C), as tested by gas chromatography-mass spectrometry. Consistently, knockdown of circMyc significantly reduced cellular triglyceride and cholesterol contents in both BT-20 and MDA-MB-231 cells (Fig. 3D, E). Moreover, lipid accumulation was notably slowed down by circMyc silencing (Fig. 3F).

### CircMyc upregulates the key lipogenic transcription factor SREBP1

**Fig. 4:**
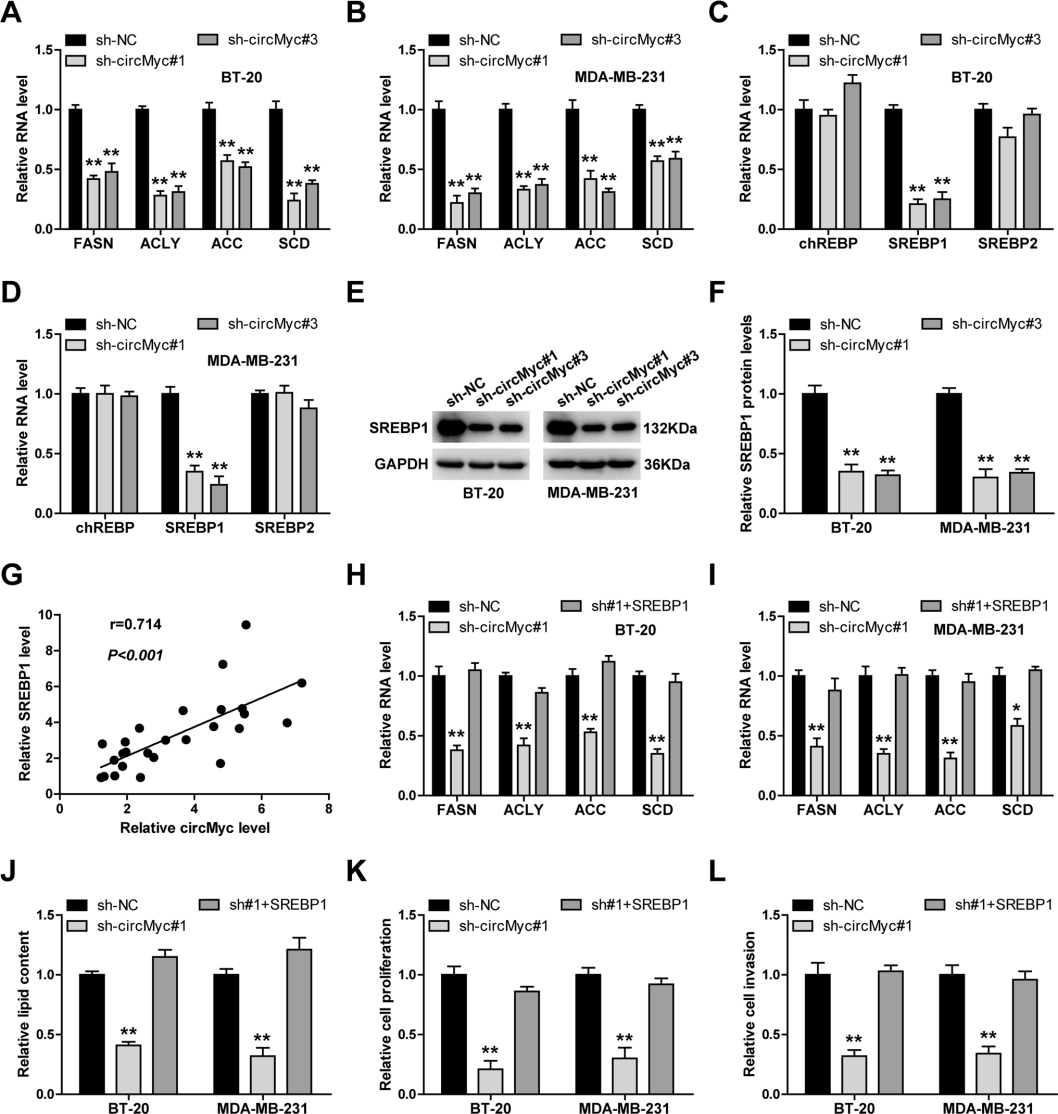
CircMyc enhances fatty acid synthesis via SREBP1. **A**–**D** qRT-PCR analysis of expression of FASN, ACLY, ACC, SCD, chREBP, SREBP1 and SREBP2 in control and circMyc-silenced BT-20 and MDA-MB-231 cells, Gene relative expression was calculated using 2^−ΔΔCt^ method, and GAPDH was used as reference control. Data were shown as the mean ± SD of at least three independent experiments carried out in triplicate. Data were analyzed by one-way ANOVA with Tukey post-hoc test. **E**, **F** Western blot testing SREBP1 protein levels in control and circMyc-silenced BT-20 and MDA-MB-231 cells. GAPDH was used as loading control. The grey density value was determined by Image J software, Data were shown as the mean ± SD of at least three independent experiments carried out in triplicate. Data were analyzed by one-way ANOVA with Tukey post-hoc test. **G** The correlation between circMyc and SREBP1 in TNBC tissues (n = 26), Data was shown as scatter diagram and analzyed by spearman correlation coefficient. **H**, **I** qRT-PCR analysis of expression of FASN, ACLY, ACC, SCD in control and circMyc-silenced BT-20 and MDA-MB-231 cells after SREBP1 overexpression. Gene relative expression was calculated using 2^−ΔΔCt^ method, and GAPDH was used as reference control. Data were shown as the mean ± SD of at least three independent experiments carried out in triplicate. Data were analyzed by one-way ANOVA with Tukey post-hoc test. **J**–**L** The effects of SREBP1 overexpression on lipid contents (tested by Nile Red staining), proliferation (tested by CCK-8 assay) and invasion (tested by Transwell assay) in circMyc-silenced BT-20 and MDA-MB-231 cells. Data were shown as the mean ± SD of at least three independent experiments carried out in triplicate. Data were analyzed by one-way ANOVA with Tukey post-hoc test. SD, standard deviation. Two-tailed **P < 0.01

As shown in Fig. 4A, B, circMyc knockdown significantly reduced the levels of lipogenic enzymes, including FASN, ACLY, ACC and SCD. Given that these lipogenic enzymes were tightly regulated by the transcription factors chREBP and SREBPs, we then tested whether circMyc affected their expression. The qRT-PCR results showed that only SREBP1 was downregulated after circMyc silencing (Fig. 4C, D). Similarly, this decrease was also observed at the protein level (Fig. 4E, F). Moreover, circMyc expression was positively correlated with SREBP1 expression in TNBC tissues (Fig. 4G). The reduced expression of above lipogenic enzymes caused by circMyc knockdown was significantly abolished by overexpression of SREBP1 (Fig. 4H, I). Besides, SREBP1 overexpression also rescued the reduced lipid accumulation, cell proliferation and invasion in two TNBC cells (Fig. 4J–L).

### CircMyc regulates SREBP1 mRNA stability via binding to HuR

**Fig. 5:**
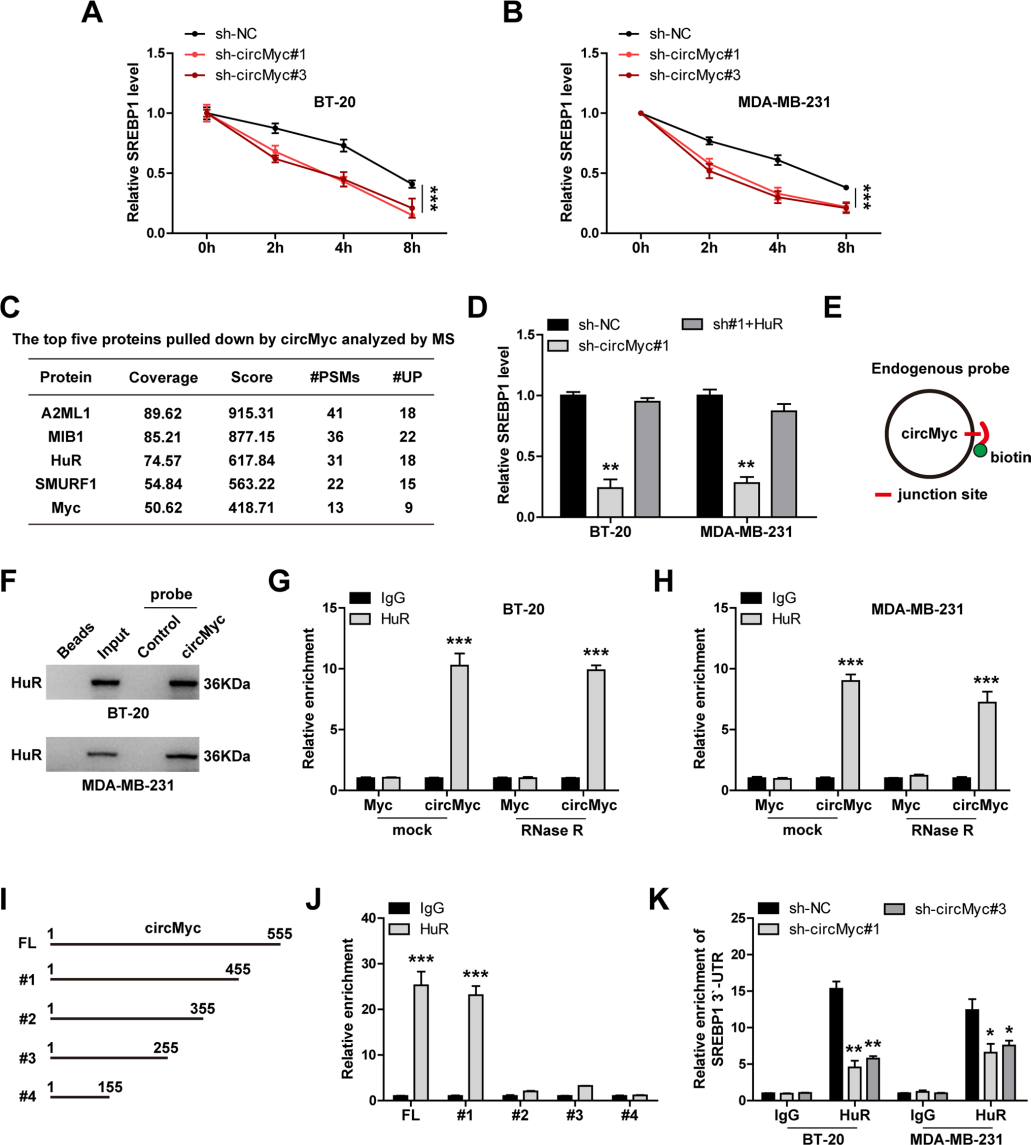
CircMyc binds to HuR. **A**, **B** qRT-PCR analysis of SREBP1 expression in control and circMyc-silenced BT-20 and MDA-MB-231 cells treated with 100µM Actinomycin D at 2 h, 4 and 8 h. Data were shown as the mean ± SD of at least three independent experiments carried out in triplicate. Data were analyzed by two-way ANOVA with Tukey post-hoc test. **C** The top five proteins pulled down by circMyc probe in BT-20 cells analzyed by mass spectrometric analysis. **D** qRT-PCR analysis of SREBP1 expression in control and circMyc-silenced BT-20 and MDA-MB-231 cells after HuR overexpression. Gene relative expression was calculated using 2^−ΔΔCt^ method, and GAPDH was used as reference control. Data were shown as the mean ± SD of at least three independent experiments carried out in triplicate. Data were analyzed by one-way ANOVA with Tukey post-hoc test. **E** The diagrammatic sketch showing the endogenous probe design targeting the junction of circMyc, followed by labelling with biotin. **F** RNA pull-down assay in BT-20 and MDA-MB-231 cells using the endogenous circMyc probe, followed by western blot analysis of HuR protein levels. **G**, **H**. RIP assay in BT-20 and MDA-MB-231 cells treated with or without 3U/µg RNase R at 37 ℃ for 20 min using 5 µg anti-HuR antibody, followed by qRT-PCR analysis of circMyc and Myc mRNA expression. IgG was used as negative control. Data were shown as the mean ± SD of at least three independent experiments carried out in triplicate. Data were analyzed by two-way ANOVA with Tukey post-hoc test. **I** The diagrammatic sketch showing the in vitro synthesis of different truncations of circMyc. **J** RIP assay in vitro using 5 µg anti-HuR or anti-IgG antibody, followed by qRT-PCR analysis of circMyc enrichment. Data were shown as the mean ± SD of at least three independent experiments carried out in triplicate. Data were analyzed by unpaired Student’s t-test. **K** RIP assay using 5 µg anti-HuR or anti-IgG antibody analyzing the effect of circMyc silencing on the binding of HuR to SREBP1 mRNA 3`-UTR in BT-20 and MDA-MB-231 cells, data were shown as the mean ± SD of at least three independent experiments carried out in triplicate. Data were analyzed by two-way ANOVA with Tukey post-hoc test. SD, standard deviation. Two-tailed *P < 0.05, **P < 0.01, ***P < 0.001

Considering that Myc is a key regulator of SREBP1[23], we then wondered whether circMyc controlled SREBP1 by Myc. As shown in Fig. 2C, Myc expression was not altered after circMyc knockdown, implying that circMyc regulates SREBP1 not by affecting Myc level. Interestingly, the half-life period of SREBP1 mRNA was significantly reduced in circMyc-silenced BT-20 and MDA-MB-231 cells (Fig. 5A, B). We then conducted RNA pull-down coupled mass spectrum analysis, the results showed that RNA binding protein HuR was significantly enriched by circMyc (Fig. 5C). Thus, we speculated that circMyc regulated SREBP1 mRNA stability via HuR. As expected, the reduced SREBP1 level caused by circMyc knockdown was rescued by HuR overexpression (Fig. 5D). The results of western blot showed that HuR protein was significantly pulled down by circMyc probe (Fig. 5E, F). Reciprocally, a large amount of circMyc was enriched by anti-HuR antibody, which was not affected by RNase R treatment (Fig. 5G, H). Then, we in vitro synthesized the truncated circMyc probe and incubated with the purified HuR protein, the results showed that 355–455 of circMyc was critical for the interaction between HuR and circMyc (Fig. 5I, J). Besides, knockdown of circMyc resulted in less HuR binding to the 3`-untranslated regions (UTR) of SREBP1 mRNA (Fig. 5K).

### CircMyc facilitates the binding of myc to SREBP1 promoter

**Fig. 6:**
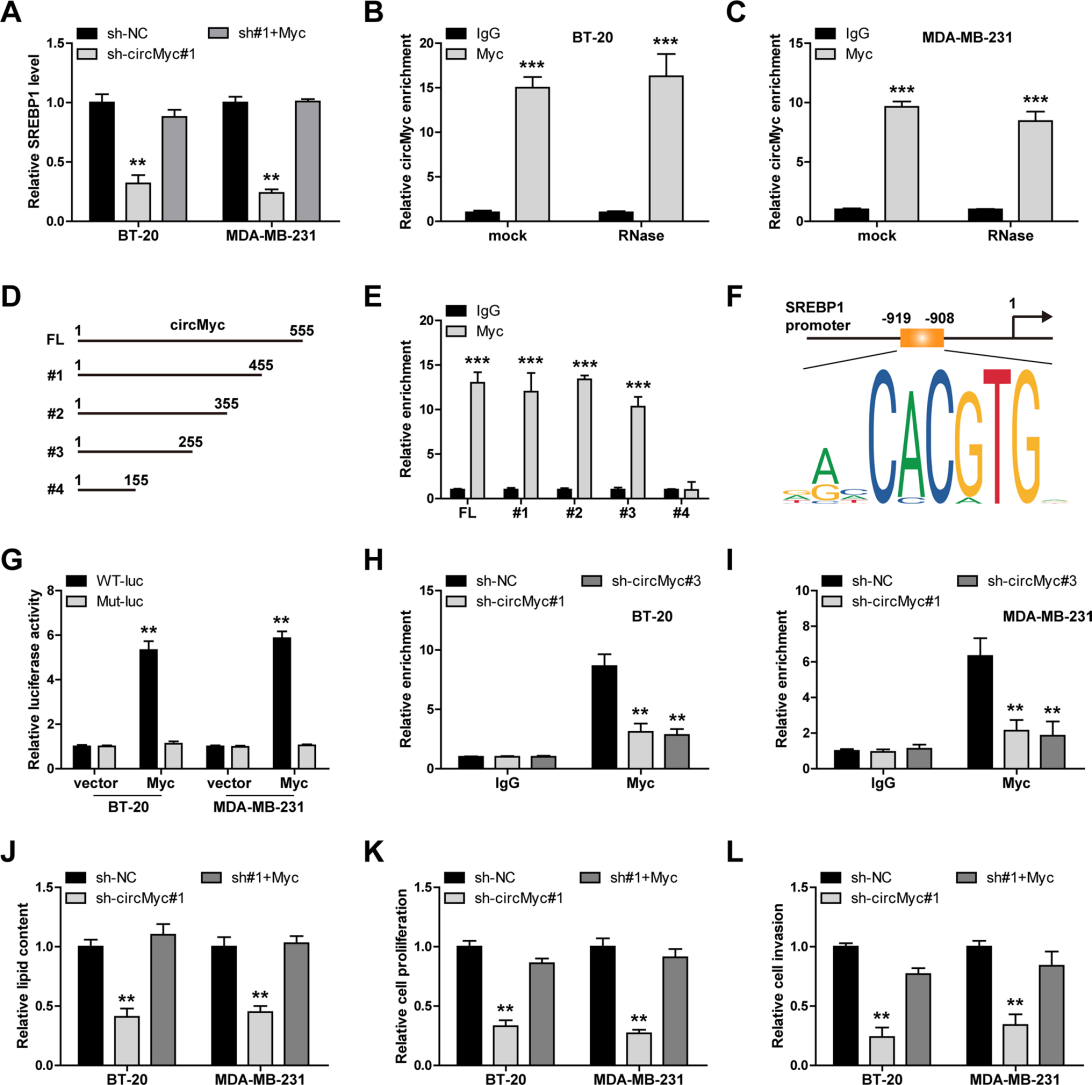
CircMyc binds to Myc. **A** qRT-PCR analysis of SREBP1 expression in control and circMyc-silenced BT-20 and MDA-MB-231 cells after Myc overexpression. Gene relative expression was calculated using 2^−ΔΔCt^ method, and GAPDH was used as reference control. Data were shown as the mean ± SD of at least three independent experiments carried out in triplicate. Data were analyzed by one-way ANOVA with Tukey post-hoc test. **B**, **C** RIP assay in BT-20 and MDA-MB-231 cells treated with or without 3U/µg RNase R at 37℃ for 20 min using 5 µg anti-Myc antibody, followed by qRT-PCR analysis of circMyc expression. IgG was used as negative control. Data were shown as the mean ± SD of at least three independent experiments carried out in triplicate. Data were analyzed by two-way ANOVA with Tukey post-hoc test. **D** The diagrammatic sketch showing the in vitro synthesis of different truncations of circMyc. **E** RIP assay in vitro using 5 µg anti-Myc or anti-IgG antibody, followed by qRT-PCR analysis of circMyc enrichment. Data were shown as the mean ± SD of at least three independent experiments carried out in triplicate. Data were analyzed by unpaired Student’s t-test. **F** The cartoon showing the binding motif of Myc at **− **919–**− **908 on SREBP1 promoter. **G** Luciferase reporter assay in BT-20 and MDA-MB-231 cells co-transfected with wild-type or mutant luciferase reporter and control or Myc overexpressing plasmid, followed by detection of SREBP1 promoter activity. Data were shown as the mean ± SD of at least three independent experiments carried out in triplicate. Data were analyzed by two-way ANOVA with Tukey post-hoc test. H, I. ChIP assay using 5 µg anti-Myc or anti-IgG antibody testing the binding of Myc on SREBP1 promoter after circMyc silencing in BT-20 and MDA-MB-231 cells. Data were shown as the mean ± SD of at least three independent experiments carried out in triplicate. Data were analyzed by two-way ANOVA with Tukey post-hoc test. **J–L** The effects of Myc overexpression on lipid contents (tested by Nile Red staining), proliferation (tested by CCK-8 assay) and invasion (tested by Transwell assay) in circMyc-silenced BT-20 and MDA-MB-231 cells. Data were shown as the mean ± SD of at least three independent experiments carried out in triplicate. Data were analyzed by one-way ANOVA with Tukey post-hoc test. SD, standard deviation. Two-tailed **P < 0.01, ***P < 0.001

As shown in Fig. 6A, Myc could elevate SREBP1 level in circMyc-silenced TNBC cells, and the mass spectrum data showed that Myc protein was pulled down by circMyc (Fig. 5D), suggesting that Myc is involved in circMyc-mediated SREBP1 regulation. The results of RIP showed that Myc protein directly bound to circMyc (Fig. 6B, C), and the further truncated assay displayed that 155–255 of circMyc was required for its interaction with Myc protein (Fig. 6D, E). The previous study have proposed that Myc bound to the − 919–− 908 motif on SREBP1 promoter, and increased SREBP1 mRNA transcription [23]. As shown in Fig. 6G, overexpression of Myc increased SREBP1 promoter activity, but this effect was blocked after mutation of -919–-908 site. Further, knockdown of circMyc significantly reduced the binding of Myc on SREBP1 promoter in these two TNBC cells (Fig. 6H, I). Functionally, the attenuated lipid accumulation, cell proliferation and invasion induced by circMyc silencing were rescued by Myc overexpression (Fig. 6J-L).

### Depletion of circMyc delays tumor growth in vivo

**Fig. 7:**
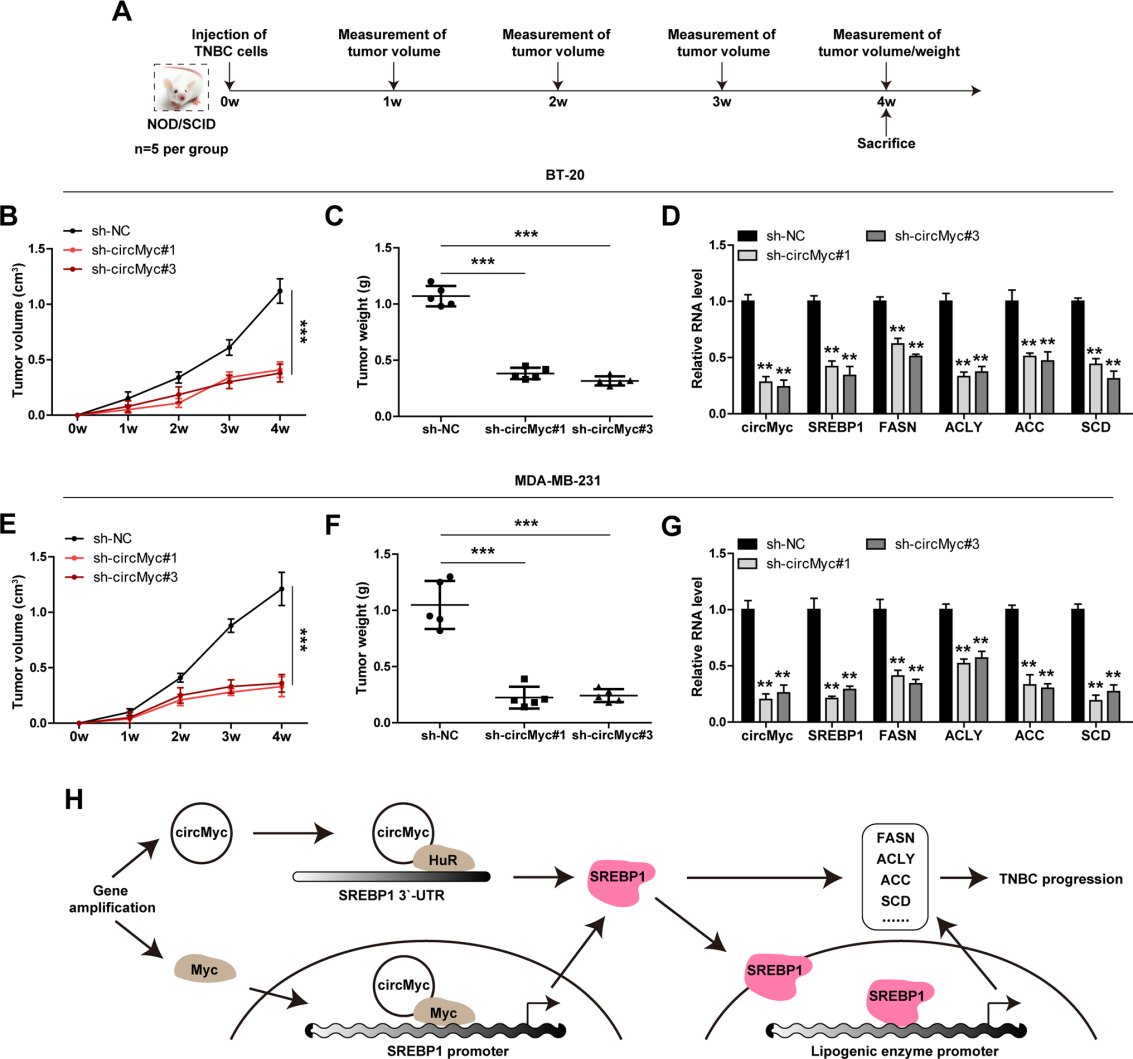
CircMyc retards tumor growth. **A** The flow chart of establishment of orthotopic tumor model via mammary fat pad injection of control and circMyc-silenced BT-20 and MDA-MB-231 cells. After four weeks, all mice were sacrificed. **B**, **C** Tumor volume and weight in NOD/SCID mice bearing control and circMyc-silenced BT-20 cells (n = 5 per group). **D** qRT-PCR analysis of expression of circMyc, SREBP1, FASN, ACLY, ACC and SCD in sh-NC, sh-circMyc#1 and sh-circMyc#3 groups. Gene relative expression was calculated using 2^−ΔΔCt^ method, and GAPDH was used as reference control, each gene was set with three technical repeat wells. Data were shown as the mean ± SD and analyzed by one-way ANOVA with Tukey post-hoc test. **E**, **F** Tumor volume and weight in NOD/SCID mice bearing control and circMyc-silenced MDA-MB-231 cells (n = 5 per group). **G** qRT-PCR analysis of expression of circMyc, SREBP1, FASN, ACLY, ACC and SCD in sh-NC, sh-circMyc#1 and sh-circMyc#3 groups. Gene relative expression was calculated using 2^−ΔΔCt^ method, and GAPDH was used as reference control, each gene was set with three technical repeat wells. Data were shown as the mean ± SD and analyzed by one-way ANOVA with Tukey post-hoc test. **H** The cartoon showing the oncogenic role of circMyc in TNBC through regulating SREBP1 via binding HuR and Myc proteins. In detail, gene amplification results in upreguation of Myc and circMyc, then, the cytoplasmic circMyc directly binds to HuR protein and increases the binding of HuR protein to SREBP1 mRNA 3`-UTR, leading to increased SREBP1 mRNA stability; On the other hand, the nuclear circMyc directly binds to Myc protein and increases the binding of Myc on SREBP1 promoter, resulting in increased SREBP1 mRNA transcription; Subsequently, the elevated SREBP1 enters into the nucleus and activates a cohort of lipogenic enzymes, leading to enhanced *de novo* lipogenesis and TNBC progression. TNBC, triple-negative breast cancer; SD, standard deviation. Two-tailed **P < 0.01, ***P < 0.001

Lastly, we tested the in vivo effect of circMyc in TNBC through establishment of orthotopic tumor model via injection of TNBC cells into the abdominal mammary fat pad (Fig. 7A). Four weeks later, all mice were sacrificed, and tumor volume and weight were significantly reduced in mice bearing circMyc-silenced BT-20 or MDA-MB-231 cells in comparison to those bearing control cells (Fig. 7B, C, E, F). Consistently, circMyc, SREBP1 and some lipogenic enzymes were markedly reduced in circMyc-silenced group (Fig. 7D, G).

## Discussion

In this study, we for the first time characterize the role of circMyc in TNBC. Recently, circMyc, like its parental gene Myc, was shown as an oncogene in various human cancer types, such as small cell lung cancer [24], acute myeloid leukemia [25], cervical cancer [26] and melanoma [27]. Likewise, our data showed that circMyc was significantly increased in TNBC, and knockdown of circMyc repressed the oncogenic phenotype of TNBC cells, suggesting that circMyc may be a pan-oncogene in human cancer. Given that Myc amplification is a common phenomenon in cancer, it is reasonable to speculate that the upregulation of circMyc is also due to the copy number variation of Myc. Such is the case, the copy number of circMyc frequently increased in human breast cancer tissues, especially in TNBC tissues, which was positively correlated with Myc amplification. Further, we found that circMyc increased de novo lipogenesis by elevating SREBP1, the well-known master regulator of lipogenic enzymes, via acting as the protein binding partner. Specifically, the cytoplasmic circMyc bound to HuR and increased HuR binding to SREBP1 mRNA 3`-UTR, reducing the decay of SREBP1 mRNA; on the other hand, the nuclear circMyc bound to Myc and increased Myc binding to SREBP1 promoter, activating SREBP1 transcription (Fig. 7H). Therefore, our findings demonstrate that circMyc promoted TNBC progression through increasing de novo lipogenesis in a SREBP1-dependent manner. The expression and role of circMyc in other malignant tumors, and whether it affects metabolic reprogramming, deserve further investigation.

CircRNA has a variety of functions, to some extent, depending on its subcellular location [28]. The cytoplasmic circRNA mainly functions as sponging miRNAs, binding to proteins and translating into functional proteins, while the nuclear circRNA mainly regulates gene transcription via interaction with some functional factors [28]. Among them, a growing body of evidence indicates that the model of circRNA-protein interaction is critical for human disease progression [29, 30]. For example, circADAMTS6 accelerated glioblastoma progression by recruiting and stabilising ANXA2 in a proteasome-dependent manner [31]. CircME1 bound to U1 snRNP and increased the transcription of its parental gene ME1, thus promoting aerobic glycolysis and sunitinib resistance of clear cell renal cell carcinoma [32]. Herein, we found that circMyc was both expressed in the cytoplasm and nucleus, and bound to a variety of proteins. Further investigations revealed that circMyc increased SREBP1 levels by binding to HuR in the cytoplasm and binding to Myc in the nucleus, respectively, suggesting that circMyc functions as protein binding partners in TNBC in different spaces. Of note, circRNA localization varies according to different stages, tissues or disease [33]. Hence, future studies need to clarify the biological processes that lead to the dynamic nuclear import and export of circMyc, which may involve some modifications, such as N6-methyladenosine (m^6^A) [28]. Moreover, we identified that 355–455 of circMyc was required for its binding to HuR, while 155–255 of circMyc was critical for its interaction with Myc. Designing some oligonucleotides to block the interactions between them may be of great significance to the metabolic reprogramming and tumor progression driven by the amplification of Myc gene.

Metabolic reprogramming is increasingly recognized as an essential hallmark of human cancer [34]. A common feature of cancer cells is their ability to rewire their metabolism to provide ATP and macromolecules needed for cell growth, division and survival [35]. The importance of fatty acid metabolism in cancer has been raised because, in addition to being structural components of membrane matrix, they are important secondary messengers and can also serve as a fuel source for energy production [36]. Emerging evidence suggests that lipogenesis is significantly enhanced in cancer cells as compared to normal cells, and some key enzymes (such as FASN, ACLY, ACC and SCD) responsible for the fatty acid metabolism are frequently overexpressed in human cancer, at least partly due to the overexpression of SREBP1 gene [37]. The transcription factor SREBP1 is capable to directly bind to the promoters of above lipogenic enzymes, resulting in transcription activation of these genes [38]. However, the upstream regulation network of SREBP1 is still largely unknown. A recent study showed that Myc can bind to the E-box motif at  − 919–− 908 on SREBP1 promoter, and increased SREBP1 transcription in hepatocellular carcinoma [23]. Herein, we found that the above phenomenon also occurred in TNBC cells, overexpression of Myc markedly increased the promoter activity of SREBP1, whereas when circMyc was silenced, this effect was disappeared. These data indicate that Myc derivative circRNA enhances the effect of Myc-driven lipogenesis and TNBC progression via regulation of SREBP1 transcription. On the other hand, circMyc increased SREBP1 mRNA stability in a Myc-independent manner, in which circMyc facilitated the binding of HuR to SREBP1 mRNA 3`-UTR. HuR, a RNA-binding protein, binds to adenine uridine (AU)-rich elements (AREs) on mRNA 3`-UTR, and blocks mRNA degradation [39]. Future research is needed to clarify the specific location of HuR binding on SREBP1 mRNA 3`-UTR, as well as the specific role played by cricMyc in this process.

Taken together, our study clearly suggest that Myc-originated circRNA drives fatty acid metabolism reprogramming and progression in TNBC via regulation of SREBP1 at transcriptional and post-transcriptional levels, which provides a promising prognostic indicator and therapeutic target for TNBC patients.

## Electronic supplementary material

Supplementary file 1 (DOC 165 kb)

Supplementary file 2 (XLS 15 kb)

## Data Availability

The datasets used and/or analyzed during the current study are available from the corresponding author on reasonable request.

## Abbreviations

TNBC: Triple-negative breast cancer
CircRNA: Circular RNA
ER: Estrogen receptor
PR: Progesterone receptor
FBS: Fetal bovine serum
SPF: Specific pathogen free
SD: Standard deviation
UTR: Untranslated regions

## References

[1] Harbeck N, Gnant M (2017). Breast cancer. Lancet.

[2] Sung H, Ferlay J, Siegel RL, Laversanne M, Soerjomataram I, Jemal A, Bray F (2021). Global Cancer Statistics 2020: GLOBOCAN estimates of incidence and Mortality Worldwide for 36 cancers in 185 countries. CA Cancer J Clin.

[3] Wesolowski J, Tankiewicz-Kwedlo A, Pawlak D (2022). Modern immunotherapy in the treatment of triple-negative breast cancer. Cancers (Basel).

[4] Singh DD, Yadav DK (2021). TNBC: potential targeting of multiple receptors for a therapeutic breakthrough, nanomedicine, and immunotherapy. Biomedicines.

[5] Chen L, Shan G (2021). CircRNA in cancer: fundamental mechanism and clinical potential. Cancer Lett.

[6] Salzman J, Circular RNA, Expression (2016). Its potential regulation and function. Trends Genet.

[7] Zhang HD, Jiang LH, Sun DW, Hou JC, Ji ZL (2018). CircRNA: a novel type of biomarker for cancer. Breast Cancer.

[8] Kristensen LS, Andersen MS, Stagsted L, Ebbesen KK, Hansen TB, Kjems J (2019). The biogenesis, biology and characterization of circular RNAs. Nat Rev Genet.

[9] Patop IL, Wust S, Kadener S (2019). Past, present, and future of circRNAs. EMBO J.

[10] Zhang F, Li L, Fan Z (2022). circRNAs and their relationship with breast cancer: a review. World J Surg Oncol.

[11] Wang S, Wang Y, Li Q, Li X, Feng X (2022). A novel circular RNA confers trastuzumab resistance in human epidermal growth factor receptor 2-positive breast cancer through regulating ferroptosis. Environ Toxicol.

[12] Wang Z, Li Y, Yang J, Liang Y, Wang X, Zhang N, Kong X, Chen B, Wang L, Zhao W, Yang Q (2022). Circ-TRIO promotes TNBC progression by regulating the miR-432-5p/CCDC58 axis. Cell Death Dis.

[13] Chen LL, Yang L (2015). Regulation of circRNA biogenesis. RNA Biol.

[14] Wang H, Gao X, Yu S, Wang W, Liu G, Jiang X, Sun D (2022). Circular RNAs regulate parental gene expression: a new direction for molecular oncology research. Front Oncol.

[15] Zhao C, Li X, Sun G, Liu P, Kong K, Chen X, Yang F, Wang X (2022). CircFOXO3 protects against osteoarthritis by targeting its parental gene FOXO3 and activating PI3K/AKT-mediated autophagy. Cell Death Dis.

[16] Wu N, Yuan Z, Du KY, Fang L, Lyu J, Zhang C, He A, Eshaghi E, Zeng K, Ma J, Du WW, Yang BB (2019). Translation of yes-associated protein (YAP) was antagonized by its circular RNA via suppressing the assembly of the translation initiation machinery. Cell Death Differ.

[17] Duffy MJ, O’Grady S, Tang M, Crown J (2021). MYC as a target for cancer treatment. Cancer Treat Rev.

[18] Llombart V, Mansour MR (2022). Therapeutic targeting of “undruggable” MYC. EBioMedicine.

[19] Fallah Y, Brundage J, Allegakoen P, Shajahan-Haq AN (2017). MYC-driven pathways in breast cancer subtypes. Biomolecules.

[20] Azim HJ, Peccatori FA, Brohee S, Branstetter D, Loi S, Viale G, Piccart M, Dougall WC, Pruneri G, Sotiriou C (2015). RANK-ligand (RANKL) expression in young breast cancer patients and during pregnancy. Breast Cancer Res.

[21] Dudekula DB, Panda AC, Grammatikakis I, De S, Abdelmohsen K, Gorospe M (2016). CircInteractome: a web tool for exploring circular RNAs and their interacting proteins and microRNAs. RNA Biol.

[22] Glazar P, Papavasileiou P, Rajewsky N (2014). circBase: a database for circular RNAs. RNA.

[23] Chen J, Ding C, Chen Y, Hu W, Yu C, Peng C, Feng X, Cheng Q, Wu W, Lu Y, Xie H, Zhou L, Wu J, Zheng S (2021). ACSL4 reprograms fatty acid metabolism in hepatocellular carcinoma via c-Myc/SREBP1 pathway. Cancer Lett.

[24] Yang X, Tao L, Xu Y, Li S, Yang W, Wang L, Zhu J (2022). CircMYC promotes proliferation, migration, invasion and inhibits apoptosis of small cell lung cancer by targeting miR-145/ matrix metallopeptidase 2 axis. Bioengineered.

[25] Zou X, Jiang M (2021). CircMYC regulates the mitochondrial respiration and cell viability via miR-516a-5p/AKT3 axis in acute myeloid leukemia. Am J Transl Res.

[26] Wang Z, Chen Y, Wang W, Wang H, Liu R (2021). circMYC promotes cell proliferation, metastasis, and glycolysis in cervical cancer by up-regulating MET and sponging miR-577. Am J Transl Res.

[27] Jin C, Dong D, Yang Z, Xia R, Tao S, Piao M (2020). CircMYC regulates glycolysis and cell proliferation in Melanoma. Cell Biochem Biophys.

[28] Liu CX, Chen LL (2022). Circular RNAs: characterization, cellular roles, and applications. Cell.

[29] Zang J, Lu D, Xu A (2020). The interaction of circRNAs and RNA binding proteins: an important part of circRNA maintenance and function. J Neurosci Res.

[30] Zheng S, Zhang X, Odame E, Xu X, Chen Y, Ye J, Zhou H, Dai D, Kyei B, Zhan S, Cao J, Guo J, Zhong T, Wang L, Li L, Zhang H (2021). CircRNA-protein interactions in muscle development and diseases. Int J Mol Sci.

[31] Zhao S, Li B, Zhao R, Pan Z, Zhang S, Qiu W, Guo Q, Qi Y, Gao Z, Fan Y, Xu H, Li M, Zhang J, Wang H, Xu J, Wang S, Wang Q, Qiu J, Deng L, Guo X, Zhang P, Xue H, Li G (2022). Hypoxia-induced circADAMTS6 in a TDP43-dependent manner accelerates glioblastoma progression via ANXA2/ NF-kappaB pathway. Oncogene.

[32] Zhang MX, Wang JL, Mo CQ, Mao XP, Feng ZH, Li JY, Lin HS, Song HD, Xu QH, Wang YH, Lu J, Wei JH, Han H, Chen W, Mao HP, Luo JH, Chen ZH (2022). CircME1 promotes aerobic glycolysis and sunitinib resistance of clear cell renal cell carcinoma through cis-regulation of ME1. Oncogene.

[33] Ebbesen KK, Kjems J, Hansen TB (2016). Circular RNAs: identification, biogenesis and function. Biochim Biophys Acta.

[34] Xia L, Oyang L, Lin J, Tan S, Han Y, Wu N, Yi P, Tang L, Pan Q, Rao S, Liang J, Tang Y, Su M, Luo X, Yang Y, Shi Y, Wang H, Zhou Y, Liao Q (2021). The cancer metabolic reprogramming and immune response. Mol Cancer.

[35] Ohshima K, Morii E (2021). Metabolic reprogramming of cancer cells during tumor progression and metastasis. Metabolites.

[36] Butler LM, Perone Y, Dehairs J, Lupien LE, de Laat V, Talebi A, Loda M, Kinlaw WB, Swinnen JV (2020). Lipids and cancer: emerging roles in pathogenesis, diagnosis and therapeutic intervention. Adv Drug Deliv Rev.

[37] Koundouros N, Poulogiannis G (2020). Reprogramming of fatty acid metabolism in cancer. Br J Cancer.

[38] Zhao Q, Lin X, Wang G, Targeting (2022). SREBP-1-Mediated lipogenesis as potential strategies for Cancer. Front Oncol.

[39] Schultz CW, Preet R, Dhir T, Dixon DA, Brody JR (2020). Understanding and targeting the disease-related RNA binding protein human antigen R (HuR). Wiley Interdiscip Rev RNA.

