# Supplementary material for: Myc derived circRNA promotes triple-negative breast cancer progression via reprogramming fatty acid metabolism
Source: Discov Oncol. 2023 May 12;14:67. doi: 10.1007/s12672-023-00679-2 (PMC10182216; doi:10.1007/s12672-023-00679-2)
Supplement: Supplementary file 1 — (DOC 165 kb) [file 12672_2023_679_MOESM1_ESM.doc]

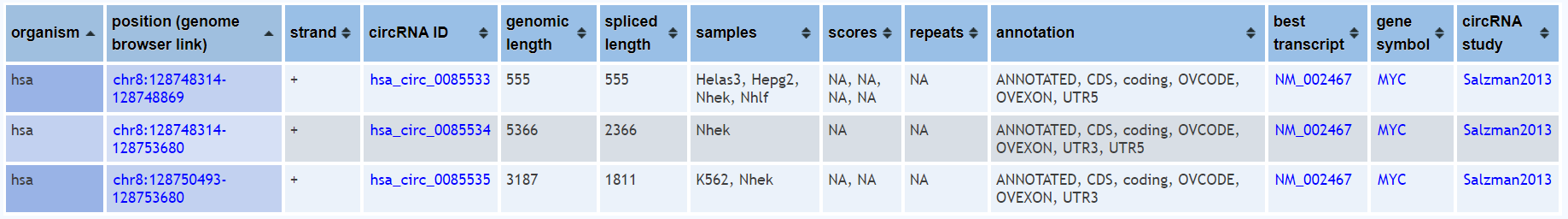


**Figure S1**. Three circRNAs derived from Myc gene in circBase database.


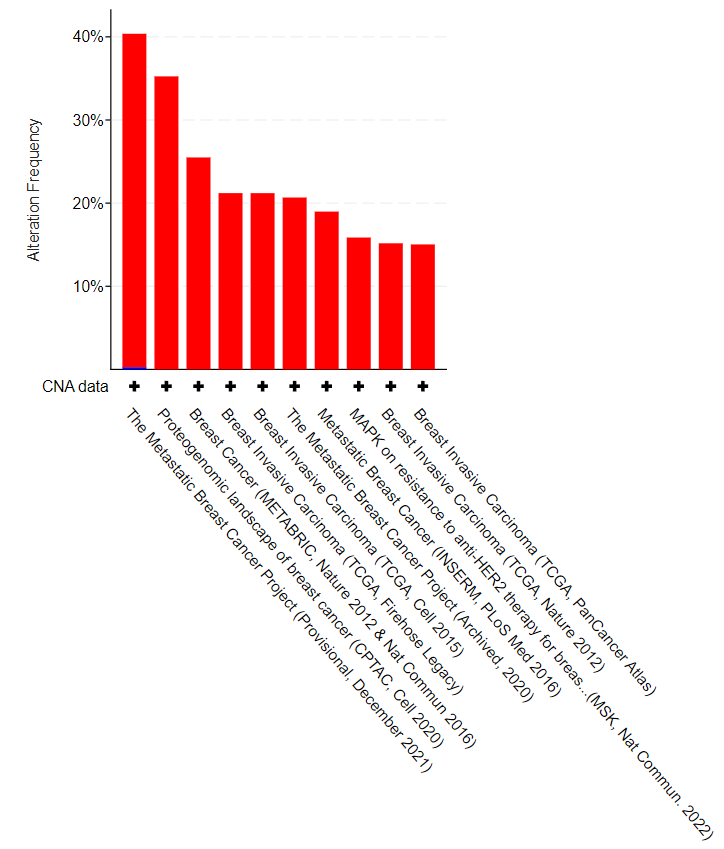


**Figure S2.** The copy number variations (CNA) of Myc gene in [cBioPortal](https://www.cbioportal.org/) database.
